# Imaging Evaluation of Dogs and Cats with Dysphagia

**DOI:** 10.5402/2012/238505

**Published:** 2012-10-31

**Authors:** Rachel E. Pollard

**Affiliations:** Department of Surgical and Radiological Sciences, Davis School of Veterinary Medicine, The University of California, Davis, CA 95616, USA

## Abstract

The current literature is reviewed in this paper regarding the application of diagnostic imaging in the evaluation of swallowing disorders of the dog. The applications of radiography, contrast radiography, and contrast videofluoroscopy are discussed with pertinent case examples provided for emphasis. The indications for image-guided interventions are also described.

## 1. Introduction

Dysphagia, or difficulty with swallowing, is a clinical symptom that may arise from a variety of diseases. Traditionally, dysphagias are classified based on the location of the abnormality (Table 1) [1, 2]. Oropharyngeal dysphagias involving the oral phase of swallowing typically result in abnormal prehension and can be diagnosed by watching the patient eat. However, oropharyngeal dysphagias affecting the pharyngeal and cricopharyngeal phases of swallowing often present with clinically similar signs such as gagging, retching, and the necessity to attempt swallowing multiple times prior to success. Myotomy or myectomy may result in clinical improvement in patients with delayed or absence of opening of the upper esophageal sphincter but animals with disorders of pharyngeal contraction will become clinically worse with surgery [3]. Moreover, animals with combined disease affecting both the pharyngeal and cricopharyngeal phases of swallowing may be poor surgical candidates.

Standard veterinary diagnostic imaging techniques can provide information regarding pharyngeal, cricopharyngeal, and esophageal anatomy [4]. However, many disease processes more specifically affect function with little or no anatomic alteration. In addition, certain anatomical alterations such as hiatal hernias are transient such that the point-in-time imaging capabilities provided by plain radiography may not capture the abnormality. Functional and transient abnormalities are particularly common in dysphagic animals. Although radiographs with or without oral barium administration may help define certain causes for dysphagia, real-time imaging techniques such as contrast fluoroscopy provide a means for visualizing esophageal function allowing for detection of subtle and transient abnormalities [4].

## 2. Normal Swallowing Anatomy and Physiology

Swallowing is a complex physiologic process that propels a bolus of liquid of food from the oral cavity to the stomach. The actions involving the lapping of liquid and prehension and chewing of food are voluntary and require coordinated movements of the tongue and jaws. Once a bolus is propelled into the pharyngeal region, the swallowing reflex occurs involuntarily requiring input from the sensory and motor branches of the trigeminal, the hypoglossal, facial, and glossopharyngeal nerves [5]. The pharynx contracts caudally against the base of the tongue to push the bolus toward the upper esophageal sphincter. Simultaneously, the epiglottis flips upward to cover the opening of the larynx to prevent aspiration. As the pharynx contracts, the paired cricopharyngeus and thyropharyngeus muscles that make up the upper esophageal sphincter relax to allow passage of the bolus into the proximal esophagus. Once the bolus has passed, the upper esophageal sphincter closes to prevent retrograde movement of the food or liquid and the epiglottis returns to its relaxed position so that normal respiration may resume.

The esophagus is a flexible tube responsible for the transport of a bolus from the upper esophageal sphincter to the stomach. Similar to the rest of the gastrointestinal tract the esophagus is a layered structure with the innermost surface being lined by mucosa. The submucosa, muscularis, and externally the adventitia are the remaining layers [4]. In the dog, the muscularis layer consists of striated muscle. In the cat, the muscularis layer consists of striated muscle cranially but changes to smooth muscle in the caudal third resulting in the characteristic “herring bone” appearance on contrast esophagography. A bolus may be transported from the proximal esophagus to the lower esophageal sphincter by primary or secondary peristaltic waves [6]. A primary peristaltic wave is a continuation of pharyngeal contraction that will propel the bolus directly to the stomach. If primary peristalsis fails then the bolus is transported to the stomach either by secondary peristalsis or with the ensuing primary peristaltic wave generated by the next bolus.

The lower esophageal sphincter is a focal narrowing of the esophagus at the junction with the stomach that is toned at rest to prevent retrograde movement of gastric contents. The sphincter is made up of a focal thickening of the muscularis layer of the esophagus, a confluence of gastric rugae at the gastroesophageal junction oriented transversely to the sphincter, and a muscular sling created by the diaphragm [4]. A short segment of the esophagus resides on the abdominal side of the diaphragm where relatively positive intraabdominal pressure compresses it. When a bolus is transported caudad by the esophagus, the lower esophageal sphincter relaxes to allow passage into the stomach.

## 3. Radiography

Survey radiography of the thorax and neck is often the first imaging step for the evaluation of the dysphagic animal. This should include three projections of the thorax (dorsoventral or ventrodorsal, right, and left lateral) and at least one lateral projection of the neck. Care should be taken to ensure that the radiograph of the neck is adequately positioned. More specifically, the lateral cervical study must not be rotated and the head should be extended as poor positioning can seriously compromise interpretation and frequently results in distortion of the anatomy.

**Figure 1:**
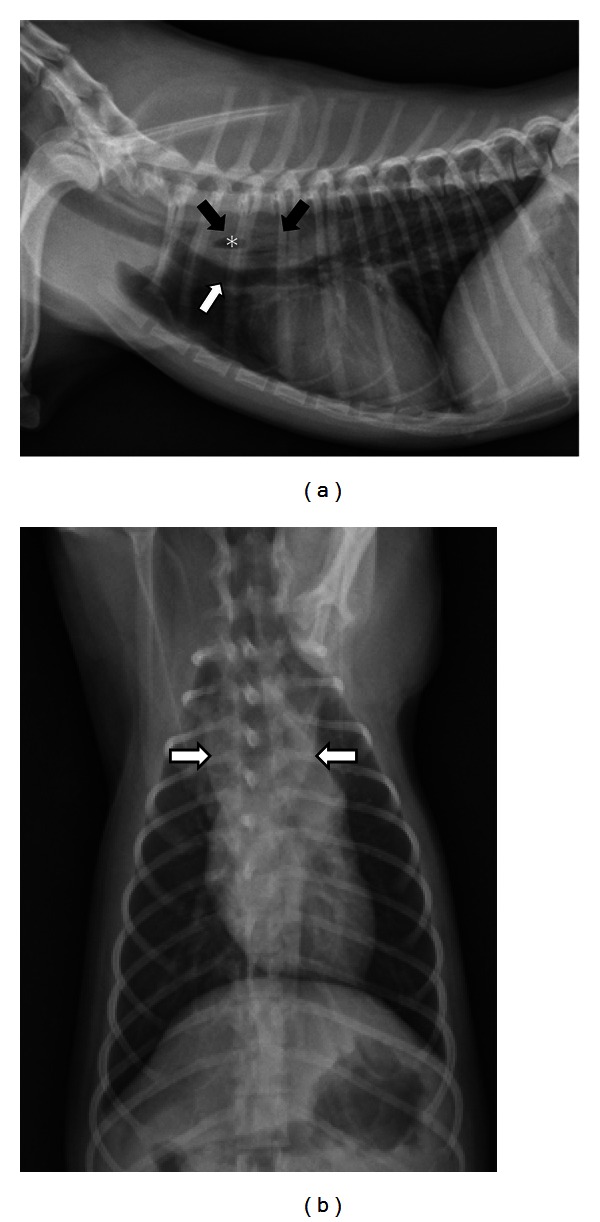
(a) A right lateral thoracic radiograph depicts a 1-year-old spayed female Yorkshire terrier with an acute onset of retching and regurgitation. A rawhide foreign body is present in the cranial thoracic esophagus (black arrows). Notice that the foreign body is difficult to see because raw hide is soft tissue density but a gas bubble in the middle is visible (∗). The distended esophagus is pushing the trachea ventrally (white arrow). There is a small amount of air in the esophagus oral to the obstruction. (b) A dorsoventral radiograph of the same dog as in (a) shows widening of the esophagus in the location of the foreign body (white arrows).

The primary purpose of survey radiographs is to rule out gross anatomic alterations that would cause dysphagia. Foreign bodies in the pharynx or esophagus may or may not be radiographically apparent [8]. Radiodense foreign bodies such as teeth [9], needles [10], and fish hooks [8] are typically readily identified with survey radiography. However, foreign bodies consisting of soft tissue dense material are harder to identify (Figure 1). Penetrating wood foreign bodies involving the pharynx and proximal esophagus have been reported in both dogs [8, 11] and cats [12] and may be hard to visualize. Radiographs obtained from dogs with penetrating wood foreign bodies in the pharynx and proximal esophagus rarely identify the stick and most commonly show soft tissue swelling and gas in the subcutaneous tissues, fascial planes, and mediastinum [8].

Pharyngeal and retropharyngeal masses of neoplastic and infectious origins can cause dysphagia but are often difficult to define using survey radiography [13–15]. However, esophageal masses may result in dysphagia and, particularly those involving the thoracic esophagus can be identified radiographically. Spirocerca lupi is a nematode that infests the canine esophagus resulting in radiographically apparent esophageal masses, caudal thoracic vertebral spondylitis, and aortic undulation [15, 16]. Transformation of Spirocerca granulomas into malignant sarcomas has been reported and also results in radiographically visible esophageal masses [17, 18]. Other types of neoplastic lesions reported to cause esophageal dysphagia include squamous cell carcinoma, leiomyoma, leiomyosarcoma, fibrosarcoma, osteosarcoma [19, 20], adenocarcinoma [21], and plasmacytoma [22].

Megaesophagus is another cause for esophageal dysphagia that can be diagnosed using survey radiography. Megaesophagus can be congenital or acquired [23]. Acquired megaesophagus is classified as idiopathic or secondary to other diseases such as myasthenia gravis [24, 25] (with or without thymic neoplasia [26, 27]), hypoadrenocorticism [7, 28, 29], hypothyroidism [30–32], tetanus [33], and dysautonomia [34, 35] to name a few. Acquired megaesophagus can also be segmental forming oral to an obstructive process such as a stricture or vascular ring anomaly [36]. Radiographic findings such as leftward deviation of the trachea and tracheal narrowing are findings associated with vascular ring anomalies that occur along with segmental esophageal dilation cranial to the heart base [36]. When diffuse megaesophagus is found, contrast imaging is typically not indicated since a cause for esophageal dysphagia has been defined and contrast administration may result in aspiration [37]. However, segmental megaesophagus should be further defined with contrast radiography in an attempt to differentiate stricture from vascular ring anomaly.

**Figure 2:**
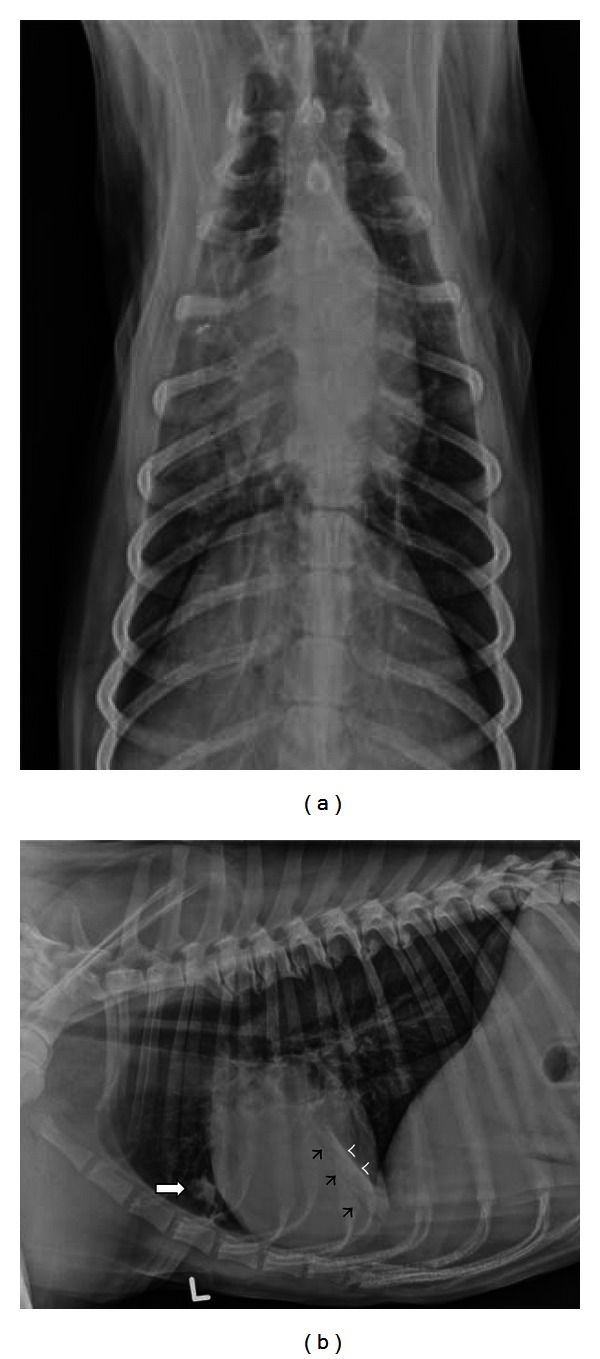
(a) A dorsoventral radiograph depicts a 4-year-old spayed female Collie with dysphagia. Alveolar infiltrates with air bronchogram formation are seen in the right cranial and middle lung lobes. (b) A left lateral radiograph of the same dog as in (a) confirms dependent pulmonary infiltrates consistent with aspiration pneumonia in the right cranial (white arrow) and right middle lung lobes. Air bronchogram formation (black arrowheads) and a lobar margin (white arrowheads) define alveolar density in the right middle lung lobe.

Survey radiographs will also help define if aspiration pneumonia is present. Aspiration pneumonia is common in dysphagic patients and typically involves the dependent aspect of the right and left cranial and right middle lung lobes. Since the right middle lung lobe is a common site for aspiration, the left lateral thoracic view is essential for complete assessment of the lung (Figure 2) [38]. When megaesophagus is identified, the presence of aspiration pneumonia has a negative association with survival time [23]. In cases where the cause for dysphagia is not clear on survey radiographs, barium contrast studies should be postponed if severe aspiration pneumonia is present, as aspiration of barium during the imaging procedure will further compromise lung function.

## 4. Contrast Radiography

If an anatomic abnormality other than megaesophagus is suspected on the survey study, contrast radiography may be indicated to help further define the origin of the problem. The type of contrast agent selected to perform contrast radiography depends on the suspected lesion type [39]. Barium paste or cream is highly viscous and may adhere best to mucosal irregularities. However, due to the viscosity of the agents, barium paste and cream may not flow well around intraluminal lesions [4]. Therefore, liquid barium (60% weight-volume) is the most common agent used for contrast radiography of the pharynx and esophagus [37]. Dosage is related to body weight such that small to medium sized dogs should receive 15 mL boluses while larger dogs should receive 20–30 mL boluses of liquid barium orally [37]. When perforation of the pharynx, cricopharynx, or esophagus is suspected, nonionic iodinated contrast agents are preferable to avoid the granulomatous reaction associated with leakage of barium into the cervical soft tissues or mediastinum [40]. Esophageal perforation should be suspected when survey radiographs identify gas in the cervical soft tissues or mediastinum but no external wounds are present. Soft tissue swelling in the cervical region may indicate abscessation subsequent to perforation should it be combined with regional emphysema. Similarly, opacification and widening of the mediastinum may accompany pneumomediastinum when perforation of the thoracic esophagus has resulted in mediastinitis [41]. In these instances, nonionic, iodinated contrast agents should be diluted 50 : 50 with sterile water and 10–15 mL boluses are administered orally [42].

Regardless of the type of contrast agent used, the animal is restrained in right lateral recumbency and liquid contrast agent is administered orally using a catheter-tipped syringe. Sedation should be avoided since tranquilizers result in significant alterations in swallowing function [42]. However, should the animal be intractable, acepromazine may be administered in dogs recognizing that rapid esophageal transit times may result particularly at higher doses [43]. Lateral and dorsoventral radiographs of the neck and thorax should be obtained during swallowing or as soon after swallowing as possible. Multiple swallows may be necessary and it is wise to obtain several views of any suspected abnormalities. When perforation is not suspected, kibble is then soaked in barium and administered orally [37]. Additional lateral and dorsoventral radiographs of the neck and thorax should be obtained. Barium soaked kibble is particularly helpful when trying to identify esophageal strictures through which liquid barium may pass unimpeded. Mixing iodinated contrast agents with kibble is not advised since iodinated agents are typically used when perforation is suspected and esophageal leakage of kibble is undesirable [42].

**Figure 3:**
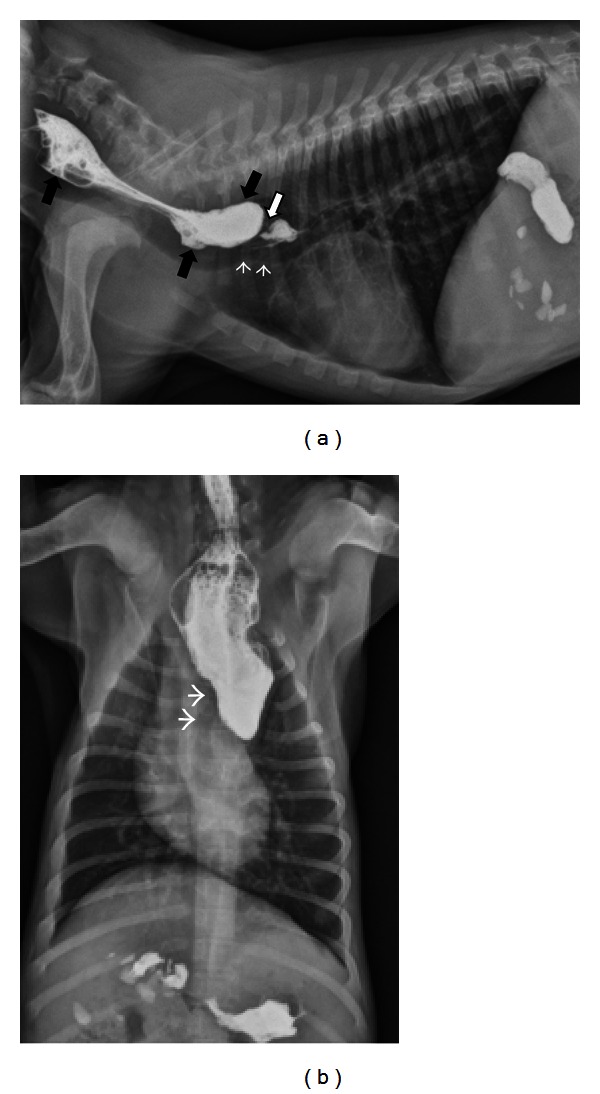
(a) A right lateral thoracic radiograph depicts a 2-month-old male Labrador presenting for regurgitation starting after weaning. Liquid barium has been administered orally immediately prior to radiography. There is a focal narrowing of the esophageal luminal diameter immediately dorsal to the heart base (white arrow). The trachea undulates and deviates ventrally in the same region (white arrowheads). Contrast is pooling is a dilated esophagus oral to the lesion (black arrows). (b) A dorsoventral radiograph of the same dog as in (a) shows contrast dilation of the cervical esophagus and an abrupt termination of the contrast column at the heart base. The trachea deviates to the left (white arrowheads) as is typically seen with persistent right aortic arch.

Specific instances where contrast radiography is particularly useful include confirmation of an esophageal foreign body suspected on survey radiographs or in the case of a young animal with a suspected vascular ring anomaly (Figure 3). A full discussion of vascular ring anomalies is beyond the scope of this paper however it is important to recognize that a multitude of types exists. Approximately 95% of vascular ring anomalies resulting in megaesophagus and esophageal dysphagia originate from persistence of the right aortic arch [36]. This can occur in isolation or in combination with other abnormal vessel development such as an aberrant left subclavian artery [44, 45]. Other infrequent types of vascular ring anomalies reported to cause esophageal dilation and dysphagia include persistent left cranial vena cava [46], double aortic arch [47], and left aortic arch with right ligamentum arteriosum [48]. Contrast esophagography can prove useful for identifying a vascular ring anomaly but often cannot distinguish between the different types [48]. Keep in mind that a single narrowed area in the esophagus may represent a peristaltic wave and strictures or vascular ring anomalies should only be diagnosed if persistent narrowing is present on several projections and abnormal dilation of the esophagus is present.

**Figure 4:**
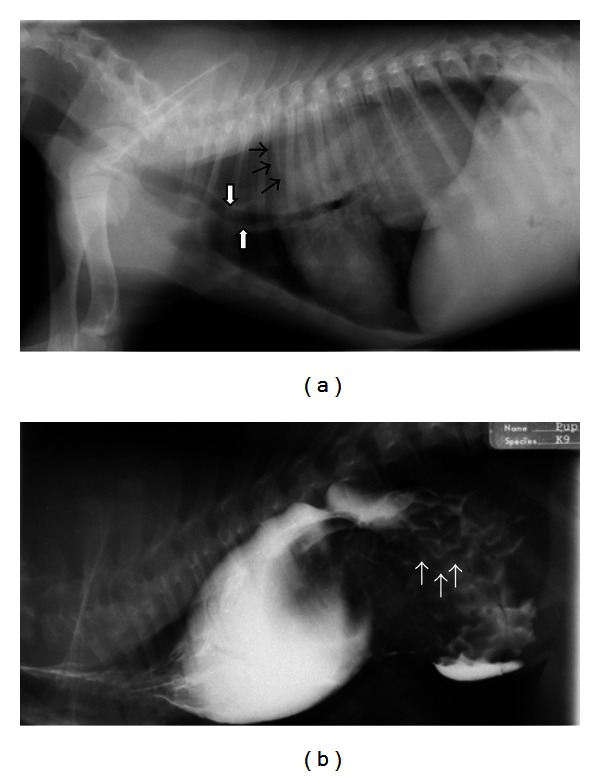
(a) A right lateral thoracic radiograph shows a 3-month-old male German Shepherd presenting for regurgitation and retching. There is gas distention of the cranial thoracic esophagus and a large soft tissue dense mass visible in the caudal dorsal thorax. A gas-soft tissue interface is seen at the cranial margin of the mass (black arrows) indicating that the mass resides within the esophagus. The trachea is displaced ventrally (white arrows). The stomach is not visualized in the abdomen. (b) A right lateral thoracic radiograph of the same dog as in (a) following the administration of liquid barium confirms that the mass is in the esophagus. There are rugal folds on the surface of the mass (white arrows) verifying the diagnosis of gastroesophageal intussusception.

Contrast radiography can also be useful when a large soft tissue mass is present in the caudal mediastinum but the origin of the mass is unclear. Contrast medium can help identify the exact location of the esophagus in the mass. Additionally, it may identify irregularity of the esophageal mucosa, which would indicate an esophageal origin for the mass. Gastroesophageal intussusception is a disorder usually diagnosed in young dogs [49–53] and cats [54] in which the stomach telescopes into the esophageal lumen. Preexisting megaesophagus may predispose to intussusception. Survey radiographs often show a heterogenous caudal mediastinal mass with proximal gas dilation of the esophagus. The gastric shadow may be absent from the abdomen. Liquid barium administration (Figure 4) will help define rugal folds on the surface of the mass and confirm esophageal obstruction [4].

## 5. Contrast Enhanced Videofluoroscopy

In people, contrast enhanced videofluoroscopy is used to evaluate and classify oropharyngeal dysphagias and functional or transient disorders of the esophagus and lower esophageal sphincter [55–61]. In companion animals, the use of contrast videofluoroscopy is limited by cost and availability. However, for certain disorders, contrast videofluoroscopy is essential to reach a definitive diagnosis as to the cause of dysphagia. It is important to recognize that, although substantial data is present in the medical literature documenting the effect of bolus size [62–66], number [67], consistency [68–72], viscosity [73–75], and body positioning during the procedure [76–79] on the outcome of videofluoroscopy in people, very little information has been generated on these topics in veterinary patients. Moreover, substantial evidence is present indicating that age delays esophageal transit times [80] and pharyngeal bolus propulsion [81, 82] in people but this has not been investigated in dogs. This is most likely related to the radiation exposure to technical staff, who must restrain the animals in order to perform research in these areas and the difficulty associated with standardizing how much food or liquid an animal swallows at any given time.

**Figure 5:**
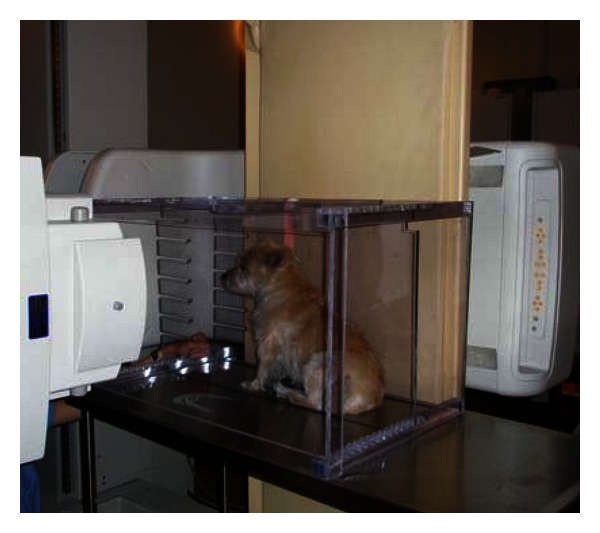
A plexiglass box is used to restrain this dog in the sitting or standing position. The X-ray source is aimed horizontally through the positioning box toward the detector located within the fluoroscopy table.

When performing an esophagram, the animal is ideally fasted for 12 hours prior to the procedure. Liquid barium (60% weight-volume) is the most common contrast agent used but, similar to contrast radiography, nonionic iodinated agents diluted 50 : 50 with sterile water can be used in cases where perforation of the pharynx or esophagus is suspected. The animal is then positioned on the fluoroscopy table. Although most institutions position the animal in right lateral recumbency, a sternal or standing position can also be used. A recent report described the differences between sternal and lateral body positioning during contrast videofluoroscopy of swallowing in the dog and found that cervical esophageal transit times were significantly delayed in laterally recumbent dogs [83]. However, a specific positioning device is necessary to minimize patient motion during sternally positioned studies (Figure 5) and, in the authors' experience, motion remains problematic during these examinations. As with contrast radiography, sedation should be avoided when performing contrast enhanced videofluoroscopy due to the inherent alteration in swallowing function that results from tranquilization.

Regardless of body position, liquid barium should be administered orally in small (5–10 mL) boluses using a catheter-tipped syringe. At least three swallows should be observed and at least one bolus should be followed all the way to the stomach. Barium-soaked kibble should also be given orally and bolus propulsion observed fluoroscopically so as to rule out a functional abnormality that allows liquid passage but affects passage of solid foods.

**Figure 6:**
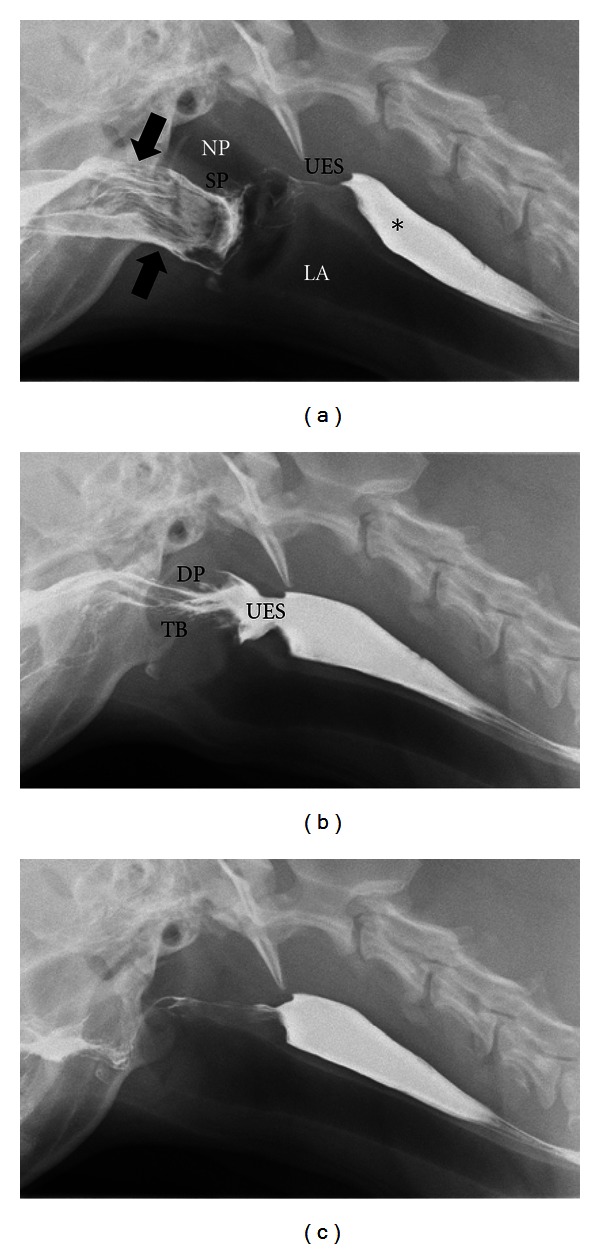
Digitally captured fluoroscopic images are shown from a 5-year-old female Golden Retriever with no evidence of dysphagia. (a) This image is taken as the dog laps barium that is placed into the mouth using a catheter tipped syringe but before swallowing is initiated. Barium contrast medium is present in the oral cavity (black arrows) with some residual barium in the proximal esophagus from the previous swallow (∗). NP = nasopharynx; LA = larynx; SP = soft palate; UES = upper esophageal sphincter. (b) The dorsal pharyngeal wall (DP) contracts ventrally to meet the tongue base (TB) and propels the liquid contrast medium caudally toward the upper esophageal sphincter (UES). The UES is wide open in coordination with caudal bolus propulsion. (c) After the bolus passes through the UES and the swallow is complete, only minimal barium remains in the oral cavity. An esophageal wave will propel the bolus from the proximal esophagus to the stomach.

Recording the study and performing frame-by-frame analysis can provide quantitative measures of swallowing function. The normal timing of the swallowing act has been reported (Figure 6) [84]. Videos are viewed frame by frame where each frame represented 1/30th of a second. The frame in which the epiglottis is observed to close over the larynx is considered as the starting point for all time measurements. From this point, frames are counted until the observation of maximal contraction of the pharynx, opening of the upper esophageal sphincter, and closing of the upper esophageal sphincter. The swallow is considered completed when the epiglottis was observed to reopen. Once the number of frames to each point in the swallow are calculated, that number is divided by 30 to obtain the number of seconds from the initiation of the swallow to each particular event within that swallow (each frame represents 1/30th of a second in the NTSC system, the analog television system used in the United States). Normal values are listed in Table 2.

The contraction of the pharynx can also be assessed using frame-by-frame analysis to calculate the pharyngeal constriction ratio [85]. From the digitized videofluoroscopic studies, a hold frame and a maximum contraction frame are selected. The hold frame is identified as a frame in which the larynx appeared at rest without rostral or caudal motion. The maximum contraction frame is identified as a frame in which the dorsal pharyngeal wall had reached its most ventral and caudal position. For the hold frame, a region of interest (ROI) is drawn around the air space beginning dorsal to the soft palate then rostrally to the hyoid apparatus and tympanic bulla, dorsally to the dorsal aspect of the pharyngeal wall, caudally along the dorsal aspect of the pharyngeal wall to the upper esophageal sphincter, and ventrally around the corniculate process of the arytenoid cartilage to include the vallecula, finally connecting the epiglottis to the starting point. The upper esophageal sphincter is specifically defined as the region in the cranial esophagus that remained the narrowest through the study [86]. For the maximum contraction frame, an ROI is drawn around any residual barium or airspace identified within the pharyngeal area. The ROI's are expressed in pixel numbers and the pharyngeal constriction ratio is calculated by dividing the number of pixels in the maximum contraction frame by the number of pixels in the hold frame. Normal pharyngeal constriction ratio in the dog is 0.15 ± 0.36 [85].

**Figure 7:**
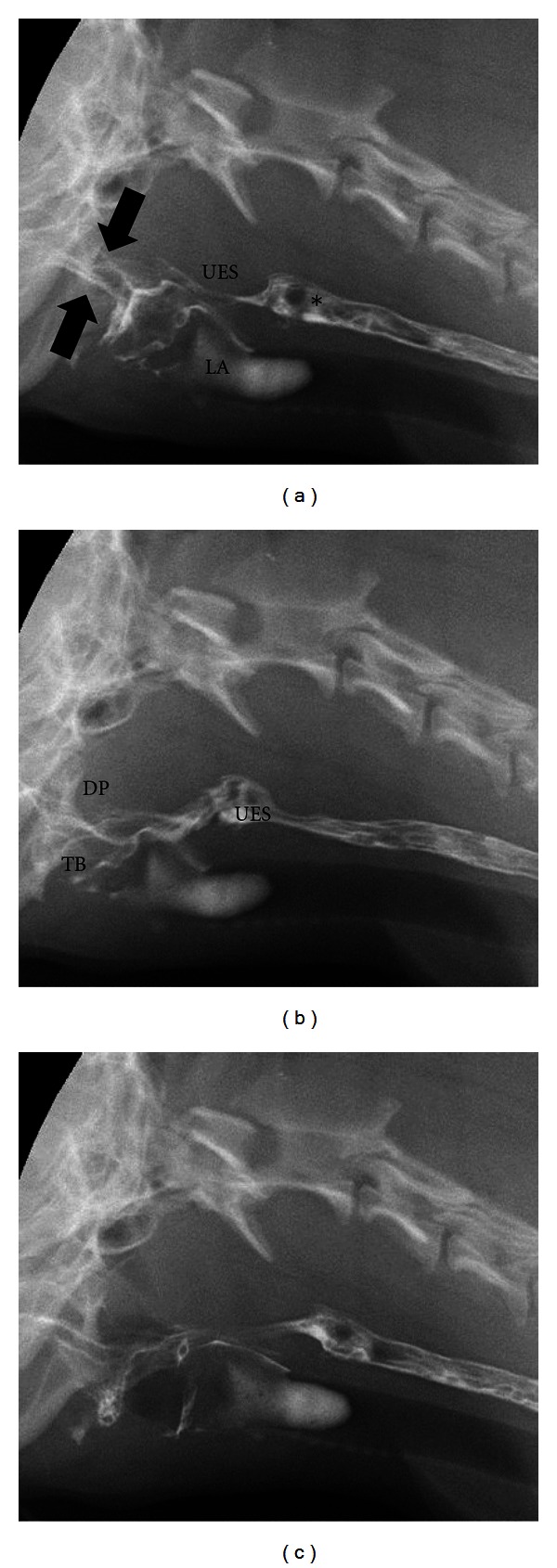
Digitally captured fluoroscopic images are shown from a 2-year-old castrated male Cavalier King Charles Spaniel with pharyngeal weakness related to immune mediated polymyositis. (a) This image is taken as the dog laps barium that is placed into the mouth using a catheter tipped syringe but before swallowing is initiated. A small quantity of barium contrast medium is present in the oral cavity (black arrows) with some residual barium in the proximal esophagus from the previous swallow (∗). Aspirated barium is also seen in the larynx (LA) and proximal trachea. UES = upper esophageal sphincter. (b) The dorsal pharyngeal wall (DP) contracts ventrally to meet the tongue base (TB) but bolus propulsion is lazy and incomplete. The upper esophageal sphincter (UES) opens in a timely manner in relation to pharyngeal contraction. (c) After the bolus passes through the UES and the swallow is complete a moderate quantity of barium remains in the oral cavity.

**Figure 8:**
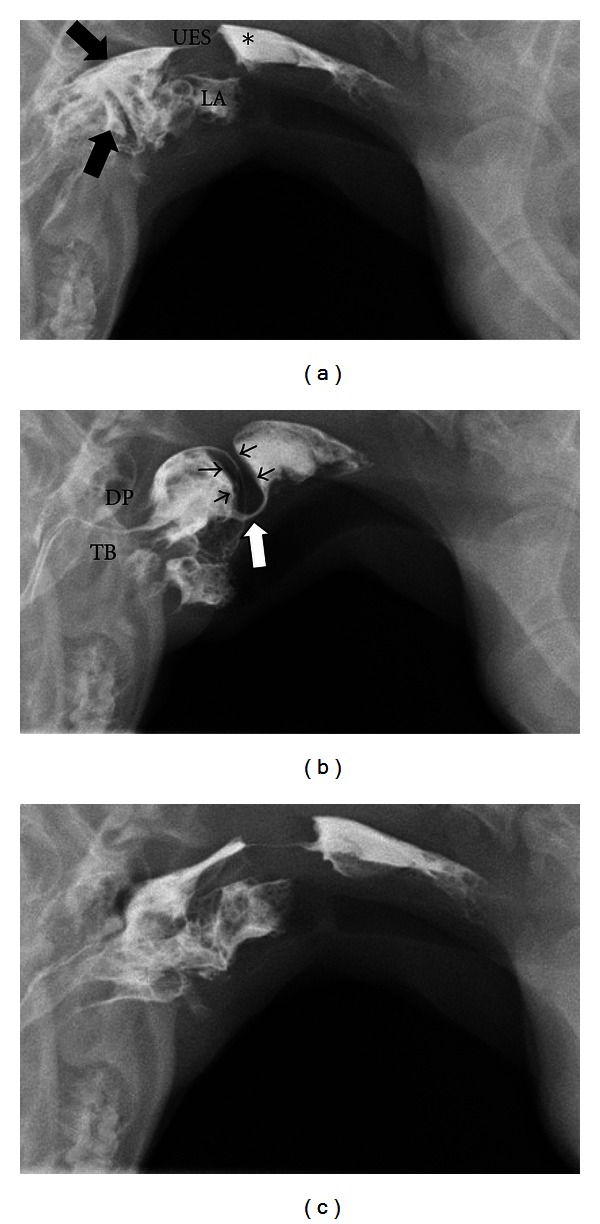
Digitally captured fluoroscopic images are shown from a 6-month-old spayed female miniature Dachshund with cricopharyngeal achalasia. (a) This image is taken as the dog laps barium that is placed into the mouth using a catheter tipped syringe but before swallowing is initiated. A moderate quantity of barium contrast medium is present in the oral cavity (black arrows) with some residual barium in the proximal esophagus from the previous swallow (∗). Aspirated barium is also seen in the larynx (LA) and proximal trachea. UES = upper esophageal sphincter. (b) The dorsal pharyngeal wall (DP) contracts ventrally vigorously to meet the tongue base (TB) but bolus passage is obstructed by a hypertrophied cricopharyngeous muscle (black arrowheads). The upper esophageal sphincter (white arrow) attempts to open in coordination with caudal bolus propulsion but the luminal diameter is extremely narrow. (c) After the bolus passes through the UES and the swallow is complete a moderate quantity of barium remains in the oral cavity.

Pharyngeal and cricopharyngeal origin dysphagias are difficult to identify and distinguish from one another without videofluoroscopic assessment and the measurements described above. Pharyngeal weakness is usually affiliated with neuromuscular diseases such as pseudorabies [87], myasthenia gravis [24], inflammatory myopathies [88, 89], congenital neurologic defects [90], and muscular dystrophy [91, 92]. It is also imperative to consider rabies in cases of pharyngeal weakness [87]. Elevation of the pharyngeal constriction ratio but normal timing in regards to opening of the upper esophageal sphincter is the videofluoroscopic hallmark of pharyngeal weakness (Figure 7) [85] (Table 3). Cricopharyngeal dysfunction can be classified as achalasia (incomplete or absent opening of the upper esophageal sphincter) or dyssynchrony (delayed opening of the upper esophageal sphincter relative to bolus presentation) [93]. Cricopharyngeal achalasia is most commonly a congenital disorder [94] the underlying mechanism behind which is unknown. Biopsy of the cricopharyngeus muscle performed on dogs suffering from this disease reveal hypertrophy, atrophy, inflammation, fibrosis, and normal muscle [95, 96]. Videofluoroscopic assessment of animals with cricopharyngeal achalasia reveals absent or minimal opening of the upper esophageal sphincter upon bolus presentation (Figure 8). Occasionally a hypertrophied cricopharyngeal muscle can be identified obstructing passage of the bolus into the proximal esophagus. Cricopharyngeal dyssynchrony can be seen in older dogs so a congenital origin is unlikely [84]. However, inheritance of dyssynchrony has been demonstrated in the Golden Retriever breed [97]. The diagnosis of cricopharyngeal dyssynchrony is made with contrast videofluoroscopy where opening of the upper esophageal sphincter is delayed resulting in elevation of pharyngeal constriction ratio [85] (Table 3). An upper esophageal sphincter opening time greater than two standard deviations from the normal mean is considered delayed/dyssynchronous [84].

Contrast videofluoroscopy is an excellent method for identification of segmental or subtle esophageal motility disorders that do not result in radiographically visible megaesophagus [98]. The causes of reduced esophageal motility are likely similar to those of megaesophagus and some propose that reduced esophageal motility is a precursor to megaesophagus [1]. Moreover, esophagitis, regardless of cause, can result in reduced esophageal motility [99, 100]. In addition to reduced motility, contrast videofluoroscopy may identify irregularities of the esophageal mucosa with prolonged mucosal adherence of barium consistent with esophagitis [1]. Certain breeds appear to be predisposed to esophageal motility disorders including the Chinese Shar Pei, Bouvier des Flandres, and some breeds of terriers [91, 98, 101]. Additionally, redundancy of the esophagus has been reported as an incidental finding where gas and/or contrast material accumulates in a deviated section of the esophagus in the thoracic inlet region of young brachycephalic breeds [4]. Age also effects esophageal motility in that young animals demonstrate reduced motility that frequently improves over time [98, 102].

**Figure 9:**
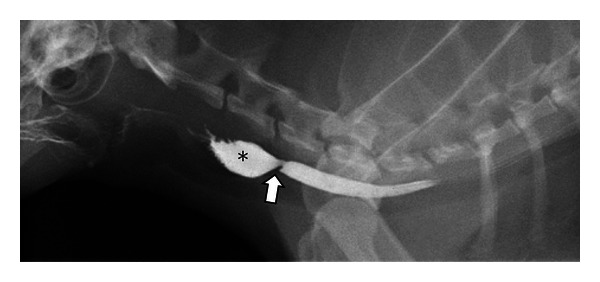
A digitally captured fluoroscopic image is shown from a 10-year-old spayed female domestic long-haired cat that presents with regurgitation following oral doxycycline therapy. Barium contrast medium has been administered and is filling the cervical esophagus. A focal narrowed region (white arrow) persists in the mid cervical esophagus. The esophageal lumen is dilated oral to the stricture (∗).

Contrast videofluoroscopy can also enhance the detection and characterization of esophageal diverticuli and strictures. Diverticuli are uncommon causes of esophageal dysphagia but have sporadically been reported in both dogs [103–105] and cats [105–107]. Diverticuli may be congenital or acquired. Acquired diverticuli can be further characterized as traction (something pulling the esophageal wall out) or pulsion (something causing increased intraluminal pressure within the esophagus) in origin [108]. Esophageal strictures can be congenital [109] but are most commonly acquired (Figure 9). In companion animals, the most common cause of esophageal stricture formation is gastroesophageal reflux associated with general anesthesia [105, 110–112]. However, esophageal stricture formation may also occur secondary to foreign bodies [103, 113], ingestion of caustic agents [111, 114], or vomiting/reflux unrelated to anesthesia [115]. Iatrogenic esophageal stricture formation has been linked to administration of doxycycline in cats [116, 117]. Esophageal neoplasms were discussed previously in this paper and may also result in stricture formation [118]. Regardless of the cause for the stricture, contrast videofluoroscopy will reveal a narrowed area of the esophagus with or without mucosal irregularity. The length of the stricture can also be defined. Dilation of the esophagus oral to the stricture may or may not be present. Some strictures are visible following liquid barium administration but many are not defined until barium soaked kibble is given.

Gastroesophageal dysphagias can result from a variety of abnormalities, many of which are transient (i.e., hiatal hernia), affect lower esophageal sphincter function, or both. Contrast enhanced videofluoroscopic esophagography can help diagnose and differentiate between these disorders. When gastroesophageal reflux or hiatal hernia is suspected, pressure may be applied to the abdomen while fluoroscopic assessment of the caudal esophagus and stomach is performed. Imaging the animal in sternal recumbency with a full stomach may also trigger reflux or hiatal herniation. In general, gastroesophageal reflux with or without hiatal herniation is difficult to identify without contrast videofluoroscopy. With videofluoroscopy, retrograde movement of contrast material from the stomach into the esophagus is visible. It is important to recognize that a small amount of reflux is normal but should be propelled back into the stomach rapidly [2].

**Figure 10:**
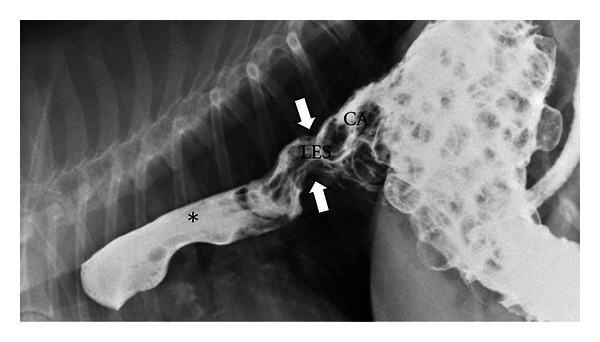
A digitally captured fluoroscopic image is shown from a 7-year-old spayed female Beagle that presents with chronic regurgitation. Liquid barium and barium soaked kibble has been administered. The lower esophageal sphincter (LES) is seen to be displaced cranial to the diaphragm and into the thoracic cavity (white arrow) and is pulling the gastric cardia (CA) with it. Liquid barium is seen to reflux from the stomach into the thoracic esophagus (∗) secondary to this Type I hiatal hernia.

Hiatal hernias involve cranial displacement of the abdominal esophagus, gastroesophageal junction, and cardia into the thoracic cavity on an intermittent or permanent basis [119, 120]. They can be classified into four distinct types. Type I is the most common and involves axial or “sliding” cranial displacement of the esophageal hiatus. Type II is a paraesophageal or “rolling” hernia. Type III is a combination of “sliding” and “rolling” of the hiatus. Type IV has been defined as a combination of Type III with herniation of abdominal organs other than the stomach [121–123]. Hiatal hernias are frequently congenital but can be seen secondary to trauma, upper airway obstruction, or tetanus [33, 124]. Contrast media in the esophagus and stomach help outline the lower esophageal sphincter making displacement more easily identified and “real-time” imaging over several minutes increases the likelihood of visualizing transient abnormalities (Figure 10). Transient disorders such as hiatal hernias and reflux may not occur during the contrast videofluoroscopic study and therefore should not be ruled out based on a negative imaging study.

Esophageal achalasia is a disorder characterized by reduced motility of the distal esophagus and failure of the lower esophageal sphincter to relax in coordination with presentation of a bolus. This disease is well documented in people [125–129] but is rare in dogs [130–133] and cats [134]. Contrast videofluoroscopy is essential to diagnosis and reveals esophageal contraction against a closed lower esophageal sphincter [130]. Retrograde movement of contrast in the esophagus results.

## 6. Image Guided Interventions

Therapeutic options for animals with esophageal dysphagias depend on the underlying etiology. While some diseases are medically managed, benign and malignant esophageal strictures prove difficult to effectively treat. Esophageal cancers are poorly responsive to chemotherapy and are in a bad location for radiation therapy due to potential radiation damage to the lung and heart [135]. Thus, they have traditionally been treated with surgical resection [18, 118, 136, 137]. Surgical resection is limited by poor visibility during surgery and the need to preserve regional anatomic structures. Dehiscence or stricture formation can occur postoperatively. Similarly, benign esophageal strictures are challenging to treat. Complications related to surgery are similar to those reported with surgical resection of esophageal tumors. Using endoscopic guidance, balloon dilation of benign esophageal strictures is now frequently performed in companion animals but success rates are variable [111, 112, 138].

**Figure 11:**
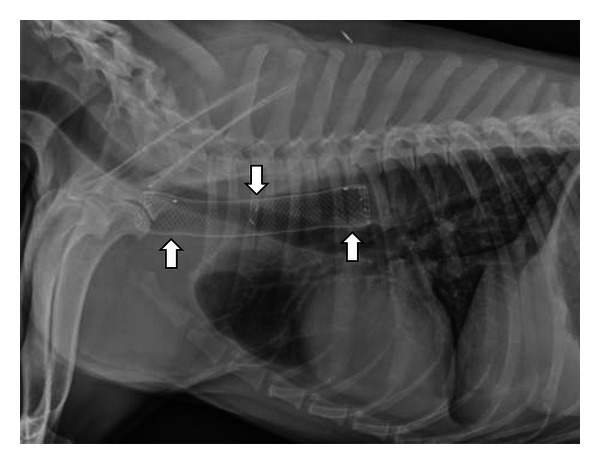
This right lateral thoracic radiograph was obtained from a 3-year-old spayed female Labrador Retriever who began regurgitating following a dental procedure. A benign stricture was diagnosed and treated with palliative stent placement when balloon dilation failed. The metallic mesh self-expanding stent is seen within the cranial thoracic esophagus (white arrows).

Palliative stenting has been reported in companion animals for the treatment of obstruction of the respiratory, urogenital, and cardiovascular systems [139–146]. Palliative stenting is well documented as a safe and effective method for treatment of esophageal obstruction in people [147–151]. Esophageal stenting for the treatment of benign strictures has been reported in dogs (Figure 11) as has palliative stenting for the treatment of esophageal neoplasia [118].

Regardless of the stricture location, placement is typically performed with the animal under general anesthesia and under fluoroscopic guidance to assure that the stent is located within the narrowed region. Mesh, self-expanding metallic stents, are most commonly used because they are radiodense, easy to deploy, flexible, and can be reconstrained and repositioned prior to complete deployment [148]. Deployment can be observed with fluoroscopy so that repositioning can occur as needed. The self-expanding mesh stents will shorten over time as they expand so this should be considered when the stent length is selected [152]. As a consequence, esophageal stents should be placed eccentrically with the greater length oral to the lesion so that some degree of caudal migration is acceptable [147]. Complication rates associated with esophageal stenting in people range from 26–52% [153, 154] and include tumor ingrowth into the stent mesh, overgrowth or granulation tissue at the stent margins, stent migration, bleeding, food bolus impaction, and esophageal injury during stent placement [150, 155].

## Figures and Tables

**Table 1:** The origin and types of dysphagia are described.

Classification	Abnormality
Oropharyngeal dysphagia	
(i) Oral phase	Difficult prehension and/or abnormal transport of bolus to tongue base
(ii) Pharyngeal phase	Abnormal transport of bolus from oropharynx to hypopharynx
(iii) Cricopharyngeal phase	Abnormal transport of bolus through upper esophageal sphincter
Esophageal dysphagia	Disorder of transport of bolus through the esophagus to the stomach
Gastroesophageal dysphagia	Abnormal transport of bolus through the lower esophageal sphincter

**Table 2:** Mean time in seconds (±standard deviation) measured from the onset of swallowing (closure of the epiglottis) in 11 healthy dogs [7].

Action	Liquid	Kibble
Maximum pharyngeal contraction	0.15 ± 0.02	0.15 ± 0.02
Upper esophageal sphincter opening	0.09 ± 0.02	0.10 ± 0.03
Upper esophageal sphincter closure	0.26 ± 0.05	0.33 ± 0.06
Epiglottic reopening	0.28 ± 0.03	0.30 ± 0.02

**Table 3:** Mean values (±standard deviation) for pharyngeal constriction ratio and time to opening of the upper esophageal sphincter in healthy dogs, dogs with pharyngeal weakness, and dogs with cricopharyngeal dyssynchrony [7].

Value	Healthy dogs (*n* = 10)	Pharyngeal weakness (*n* = 11)	Cricopharyngeal dyssynchrony (*n* = 4)
Pharyngeal constriction ratio	0.15 ± 0.36	0.60 ± 0.28	0.61 ± 0.22
Upper esophageal sphincter opening (seconds)	0.07 ± 0.00	0.07 ± 0.01	0.28 ± 0.04
